# Daptomycin-Induced Posterior Reversible Encephalopathy Syndrome (PRES)

**DOI:** 10.1155/2019/8756932

**Published:** 2019-02-24

**Authors:** A. Bitar De Zayas-Enriquez, C. Soper

**Affiliations:** Croydon University Hospital, 530 London Road, Croydon CR7 7YE, UK

## Abstract

**Aims:**

To present a 60-year-old female patient who manifested clinical and radiological features of posterior reversible encephalopathy syndrome (PRES) following the administration of Daptomycin for glycopeptide-resistant Enterococcal urinary tract infection.

**Material:**

Case report.

**Method:**

Posterior reversible encephalopathy syndrome was diagnosed in our patient following the administration of Daptomycin based on clinical suspicion as well as brain CT and MRI imaging.

**Results:**

The temporal association between the initiation of Daptomycin and the onset of PRES is highly suggestive of causality, and this is further supported by clinical and radiological resolution after Daptomycin was withdrawn.

**Conclusion:**

This is the first report of Daptomycin-induced posterior reversible encephalopathy syndrome.

## 1. Introduction

Posterior reversible encephalopathy syndrome (PRES) is a disorder diagnosed based on symptoms, seizures being the most common, and brain MRI features of bilateral hyperintensities predominantly in the occipitoparietal regions [1]. It has been associated with multiple triggers, most commonly abrupt hypertension, renal failure, immunosuppressive therapy, eclampsia, transplantation, autoimmune disease, and infections [2], particularly with gram-positive organisms [3], as well as several drugs [4–7]. The management consists of withdrawing the suspected trigger and treating hypertension aggressively [1, 2]. The majority of cases of PRES show very good short-term and long-term outcomes [1].

We report the first documented case of PRES in association with Daptomycin, a cyclic lipopeptide used mainly for the treatment of* Staphylococcus aureus* and vancomycin-resistant enterococci.

## 2. Case Presentation

We present a 60-year-old white British female patient, who was admitted to the hospital with unilateral ankle pain and swelling, stage-3 acute kidney injury (AKI), and haematuria. She had recently been discharged from hospital, after being treated for a urinary tract infection (UTI) and AKI.

On admission, the patient was initially treated with IV fluids and started on oral Prednisolone at 25 mg daily by the Rheumatology team, for what they suspected to be a flare-up of her known rheumatoid arthritis, after a deep vein thrombosis (DVT) was ruled-out on venous ultrasound-Doppler.

Extensive investigations were initiated to diagnose the aetiology of the AKI. She was also started on a broad-spectrum antibiotic (Tazobactam + Piperacillin) in-view of positive urine dip and microscopy, and urine culture later grew Glycopeptide-resistant* Enterococcus sp.*, for which Daptomycin at 4 mg/Kg was initiated, based on microbiologist advice. Prednisolone had been discontinued the day before.

Within fifty minutes of receiving the first dose of Daptomycin, the patient suffered from two tonic-clonic seizures which were five minutes apart, these lasted approximately two minutes each, and both terminated spontaneously. Further neurological examination revealed no focal deficit. A single, further, generalised seizure occurred 2 hours later for a few minutes, but no others were observed afterwards.

**Figure 1:**
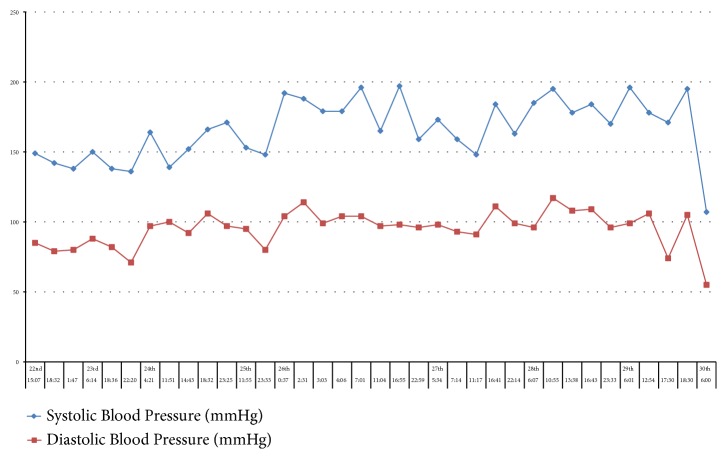
Patient's blood pressure in the week preceding the onset of seizures.

Her mean arterial pressure on that day was 130 mmHg and her oxygen saturations ranged between 94 and 97%. A plot of her elevated BP for the preceding week is shown in Figure 1.

Her haemoglobin was 105g/l, urea was 13.8 mmol/L, creatinine was 228 micromol/L, eGFR by MDRD was 19ml/min, and C-reactive protein was 14 i.u., and her liver function tests, calcium, and phosphate were normal.

**Figure 2:**
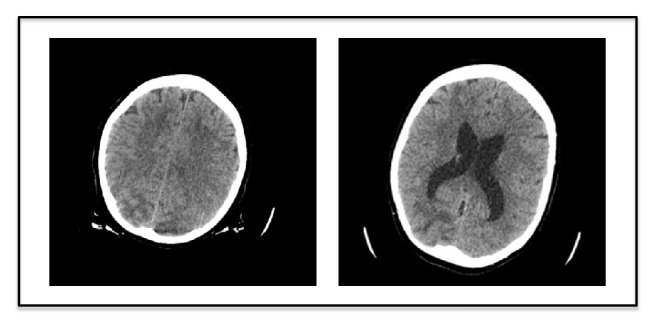
Brain CT-scan showing widespread bilateral asymmetrical hypodensities in the cortex and subcortical regions.

A CT-scan of the head, displayed in Figure 2, showed an area of low density in the occipital lobes on both sides sparing the overlying cortex and involving the underlying white matter. It also showed a further area of low density in the right frontal region. The differential diagnosis provided by the Radiologist included ischaemic changes, posterior reversible encephalopathy syndrome (PRES), or infective changes, the latter was reported as least likely.

The patient had been persistently hypertensive for at least forty-eight hours prior to the onset of seizures, without any abrupt elevations in blood pressure. She was started on a Labetalol infusion and transferred to ITU for intubation/ventilation, and a phenytoin infusion was initiated as she suffered from a third seizure whilst in the CT department. Daptomycin and Tramadol were then stopped; all other medication was continued.

Brain MRI venogram confirmed symmetrical white matter signal changes within the occipital lobes bilaterally without diffusion restriction, with more patchy white matter changes superiorly within both frontal lobes which were more pronounced on the right.

EEG showed frequent brief runs of generalised semirhythmical slow activity at times with sharp components throughout the recording, which could be postictal or related to diffuse cerebrovascular disease.

Brain MRI was repeated three weeks later and showed substantial resolution in the changes described on the previous scan with modest nonspecific residual changes only.

The patient had a few partial seizures thereafter, but eventually she was successfully weaned off antiepileptics and was discharged from hospital.

Her past medical history included hypertension, chronic obstructive pulmonary disease, pulmonary embolism, diabetes mellitus, rheumatoid arthritis, dyserythropoiesis probably secondary to disease-modifying anti-rheumatic drugs, recurrent UTI's, and depression. She was on the following regular medications: aspirin, simvastatin, metformin, leflunomide, hydroxychloroquine, epoetin, folic acid, solifenacin, chlorphenamine, omeprazole, ranitidine, quinine sulphate, cocodamol, pregabalin, tramadol, citalopram, and inhalers, all from admission, eight days before. She had no known drug allergies. She was a current smoker with history of over 30 pack-year smoking.

## 3. Discussion

Tonic-clonic seizures are the most common symptom of PRES, which our patient manifested shortly after receiving the first dose of Daptomycin. Brain imaging then supported the clinical suspicion of PRES with white matter signal changes predominantly in the occipital lobes, with major reversal on subsequent imaging.

Other risk factors for PRES also contributed towards the diagnosis: female gender, elevated blood pressure, acute kidney injury on admission, longstanding treatment with epoetin, smoking, infection with gram-positive organism, and history of autoimmune disease (rheumatoid arthritis); however these had been present at least for some days and were improving; no other immediate triggers were evident [1, 2].

The pathogenesis of PRES remains unclear but the following mechanisms have been postulated: cerebral blood flow autoregulatory failure, cerebral ischaemia as a result of focal vasoconstriction, and endothelial dysfunction, particularly in preeclampsia, cytotoxic therapy, uraemia, sepsis, and metabolic disturbances.

Our patient had chronic hypertension and her blood pressure was persistently high at a stable level for over forty-eight hours preceding the onset of seizures (see Figure 1), without any abrupt elevations to correlate with the onset of PRES, although up to 30% of cases of PRES have been described in the context of mildly elevated blood pressures where the upper limit of cerebral autoregulation was not reached [1].

Our patient had been receiving regular Erythropoietin prior to admission, started by a haematologist for macrocytic anaemia diagnosed on bone marrow aspirate as mild dyserythropoiesis probably secondary to disease-modifying anti-rheumatic drugs. Our patient received her regular dose of epoetin on the day she developed PRES, about three hours prior to the onset of seizures. Epoetin is known to induce or exacerbate hypertension, thereby contributing to cerebral dysregulation and PRES [8].

In addition, our patient presented with stage 3 acute kidney injury, which could have contributed to PRES, and the underlying mechanism is thought to be uremia-induced endothelial dysfunction [8, 9]. However, the AKI had been steadily improving over the few days that preceded the onset of seizures. She also had rheumatoid arthritis and was being treated for a gram-positive infection, both potential contributing factors for PRES [3].

The administration of Daptomycin is the only variable that immediately preceded the onset of seizures. Daptomycin is a cyclical lipopeptide; its bactericidal effect is reportedly mediated by increasing cell membrane permeability to ion flow [10]. One favoured mechanism for this has been by conjugation with phosphatidylglycerol, a lipid component rich in gram-positive bacterial cell walls against which it is active and some gram-negative bacteria [11]. Phosphatidylglycerol is less abundant but also present in mammalian cells and mitochondria, where it is precursor for the synthesis of cardiolipin and other lipid components [12]. It is particularly abundant in pulmonary surfactant. Daptomycin's datasheet reports its association with tremor, rigors, less commonly rhabdomyolysis, and neuropathy [13]. It demonstrates favourable CNS penetration in adults [14]. In dogs, in supratherapeutic regimes it has caused twitching, muscular rigidity, and muscular weakness [13]. It also has immune modulating effects [15]. We speculate that its affinity for lipid membrane components alters endothelial cell wall permeability and neuronal and myocyte function, heightening the risk of PRES in our patient. Its near entire renal excretion has raised concerns both about higher rates of toxicity due to a larger unbound fraction in patients with severe renal impairment and specifically of creatine kinase elevation in high dose regimes [16, 17]. Dose adjustment in renal disease is required [17]. Increased permeability of the blood-brain barrier in renal impairment has been reported to facilitate the penetration of Daptomycin into the CNS, thus increasing its exposure [8, 9].

## 4. Conclusion

To our knowledge, a close association between Daptomycin and PRES has not been published to date, and we would recommend vigilance in the use of Daptomycin in patients at risk and consideration of its immediate discontinuation, if a patient develops signs or symptoms of PRES.
